# Supplementary material for: A systematic review of the application of machine learning in the detection and classification of transposable elements
Source: PeerJ. 2019 Dec 18;7:e8311. doi: 10.7717/peerj.8311 (PMC6967008; doi:10.7717/peerj.8311)
Supplement: Supplemental Information 1 — Abbreviations: Random Forest (RF), Decision Trees (DT), Bayesian Networks (BN), Neural networks (NN), Hidden Markov Model (HMM), Support Vector Machine (SVM), Naïve bayes (NB), Logistic Regression (LR), K-Nearest Neighbors (KNN), Self-Organizing Map (SOM), Principal Component Analysis (PCA), Multi-Layer Perceptron (MLP), first-order random forests (FORF). [file peerj-07-8311-s001.docx]

| Publication | Data Source | Task | ML algorithm | Parameters | Framework or language | Learning method | Reference |
| --- | --- | --- | --- | --- | --- | --- | --- |
| P2 | Numerical and categorical features based on coding regions | Detect LTR Retrotransposons at super-family level | RF | Minimum number of examples in a leaf was 5 and number of trees was 100. No pruning was used, RF based on FORF | ACE | Supervised | [1] |
| P3 | Numerical and categorical features | Classify LTR Retrotransposons at lineage level | DT, BN and lazy algorithms | Default in Weka implementation | Weka | Supervised | [2] |
| P4 | Numerical and categorical features | Improve the detection and classification of TEs | NN, BN, RF, DT | 500 training cycles and 0.3 of learning rate for NN; DT based on c4.5 algorithm | Weka | Supervised | [3] |
| P5 | RNA | Detect boundary sequences of mobile elements | HMM, SVM | Linear kernel for SVMs |  | Supervised | [4] |
| P6 | RNA | Detection of cancer-related long non-coding RNA | RF, NB, SVM, LR and KNN | Random Fores:n_estimators=100, criterion='gini'; Naïve bayes:alpha = 5; SVM:kernel = 'rbf', C= np.logspace(-2, 10 ,13), gamma= np.logspace(-9, 3, num = 13); Logistic regression: C=10, KNN: (n_neighbors = 3 | Scikit-learn | Supervised | [5] |
| P8 | Z-score features, representing chromosome arm gains and losses. | Detection of aneuploidy | SVM | radial basis kernel and default parameters | R (e1071 package) | Supervised | [6] |
| P10 | K-mer frequencies and frequencies of certain patterns | Distinguishing Endogenous Retroviral LTRs from SINEs | RF | Default in randomForest package in R | R (randomForest Package) | Supervised | [7] |
| P11 | Dinucleotide frequencies | Identification and clustering of RNA structure motifs | Density-based clustering | Calculated by a binary classifier | Java | Unsupervised | [8] |
| P12 | DNA and categorical features | Automatization of the process of extracting discriminatory features for determining functional properties of biological sequences | Evolutionary Feature Construction and Evolutionary Feature Selection |  | Java | Unsupervised | [9] |
| P14 | Numerical features | Analysis of mutants | RF | Default parameters | Scikit-learn | Supervised | [10] |
| P15 | Insertion sites | Identiﬁcation of potential insertion sites of mobile elements | SVM |  |  | Supervised | [11] |
| P16 | Numerical features | Identification of somatic LINE-1 insertions | LR | Default in caret package in R | R | Supervised | [12] |
| P17 | Numerical features, ARN mononucleotides, dinucleotides and trinucleotides frequencies, Fickett score | Identification of most informative features of long non-coding transcripts | 11 different feature selection approaches, SVM , RF, and NB | SVM: Gaussian radial kernel and C=1; | R | Supervised | [13] |
| P19 | Numerical and categorical features | Improve the detection and classification of TEs | NN, BN, RF, DT | 500 training cycles and 0.3 of learning rate for NN; DT based on c4.5 algorithm | Weka | Supervised | [14] |
| P21 | K-mer frequencies | Classify repetitive sequences | SVM |  |  | Supervised | [15] |
| P22 | Numerical features | Prediction of microRNA precursors | SVM | Kernel radial basic function | C++ (libsvm library) | Supervised | [16] |
| P24 | DNA | Detecting repeats de novo | HMM |  | C++ | Supervised | [17] |
| P26 | K-mer frequencies | Classify TEs using hierarchical | DT, RF, NB, KNN, MLP, SVM | MLP: one hidden layer containing 200 neurons and using a logistic function; DT based on c4.5 algorithm | Scikit-learn | Supervised | [18] |
| P27 | Oligomer frequencies | Classify TEs | SVM | Gaussian kernel | C++ (libsvm library) | Supervised | [19] |
| P28 | Numerical features based on structure | Identify sequence motifs conserved in each of the ﬁve major TIR super families. | NN, KNN, RF, and Adaboost | NN: 30 hidden layers and 100 neurons per layer, activation function=tanh, Learning rate=0.001; RF: n_stimator=200, criterion=gini, max_depth=None, max_features=auto; KNN: n_neighbors=20,weights=distance, algorithm=ball_tree; Adaboost: criterion=gini, max_depth=50, max_features=auto, n_estimator=20 | Sklearn | Supervised | [20] |
| P30 | Numerical features and k-mer frequencies | piRNA prediction | SVM | Kernel: custom Gaussian | C++ (libsvm library) | Supervised | [21] |
| P31 | Aligned genomes and binary representation (1 for mismatches and 0 for matches) | recognition of local relationship patterns | HMM, SOM | HMM penalty: 150; SOM neurons: 800 | C++ | Unsupervised | [22] |
| P32 | Numerical features | Compare multiple transposon insertion sequencing studies | PCA | single value decomposition, “centered” set to off | Matlab | Unsupervised | [23] |
| P33 | Numerical and categorical features, nucleotide frequencies | Classify the precursors of small non-coding RNAs | RF |  | R | Supervised | [24] |
| P34 | Normalized numerical and categorical features | Prediction of transcriptional effects by intronic endogenous retroviruses | MLP NN | 3 Layers: input layer with four neurons, a hidden layer of three neurons mutually connected to all the other neurons in both the input and output layers; the last layer is the output layer, which consists of only a single neuron as the output node. | R (neuralnet package) | Supervised | [25] |
