# Supplementary material for: A systematic review of the application of machine learning in the detection and classification of transposable elements
Source: PeerJ. 2019 Dec 18;7:e8311. doi: 10.7717/peerj.8311 (PMC6967008; doi:10.7717/peerj.8311)
Supplement: Supplemental Information 2 [file peerj-07-8311-s002.docx]

| 3 hidden layers (one for auto-encoder with L1 regularization), back-propagation | Reference |
| --- | --- |
| 2 hidden layers with each layer including 500 hidden units | [26] |
| from no hidden layers up to three hidden layers with maximally 256, 128, and 64 units in the first, second, and third hidden layers, l2-regularization, ReLU activation function | [27] |
| Convolution, Rectify, Pool, NN | [28] |
| 1. Convolution layer (320 kernels. Window size: 8. Step size: 1.) 2. Pooling layer (Window size: 4. Step size: 4.) 3. Convolution layer (480 kernels. Window size: 8. Step size: 1.) 4. Pooling layer (Window size: 4. Step size: 4.) 5. Convolution layer (960 kernels. Window size: 8. Step size: 1.) 6. Fully connected layer (925 neurons) 7. Sigmoid output layer | [29] |
| 3 convolution layers 2 two layers of fully connected hidden nodes, ReLU activation function | [30] |
| Convolution layers, rectification layers (ReLU), pooling layers and fully connected layers. | [31] |
| Convolution layers, followed by dilated convolution layers and a final convolution layer. All layers applied batch normalization, rectified linear units, and dropout. | [32] |
| Convolution, Pool, Convolution, Pool, Convolution, Convolution, Dense layer. All layers are followed by a batch normalization layer then a ReLU activation | [33] |
| Two convolutional and pooling layers and one fully connected hidden layer, ReLU activation function | [34] |
| Input (4bases x 2000bp), 1. Convolution (4 -> 320, kernel size=8), 2. ReLU, 3. Convolution (320 -> 320, kernel size=8), 4.ReLU, 5. Dropout (Probability=0.2), 6. Max pooling (pooling size=4), 7. Convolution (320 -> 480, kernel size=8). 8. ReLU, 9. Convolution (480 -> 480, kernel size=8), 10. ReLU, 11. Dropout (Probability=0.2), 12. Max pooling (pooling size=4), 13. Convolution (480 -> 960, kernel size=8), 14. ReLU, 15. Convolution (960 -> 960, kernel size=8), 16. ReLU, 17. Dropout (Probability=0.2), 18. Linear (101760 -> 2003), 19. ReLU, 20. Linear (2003 -> 2002), 21. Sigmoid, Output (Size: 2002 epigenomic features) | [35] |
| 1. Convolutional, 128 filters (4 × 13), relu activation, 0.15 dropout probability, 2. Convolutional, 128 filters (1 × 13), relu activation, 0.15 dropout probability, 3. Convolutional, 128 filters (1 × 13), relu activation, 0.15 dropout probability, 4. Fully connected layer, 64 hidden units, relu activation, no dropout, 5. Linear output layer, 1 output unit | [36] |
| Variable number of convolutional/max-pooling stacks followed by a variable number of dense layers interspersed by dropout layers. | [37] |
| Input layer (14x14), 1. Convolutional (12x12x6), 2. Sub-sampling layer (6 feature maps), 3. Convolutional (12x10x10), 4. Sub-sampling layer (12 feature maps), 5. fully connected NN, Output layer (4) | [38] |
| Bidirectional Gated Recurrent Network, the embedding layer is fully connected and used ReLU as activation function | [39] |
| Bidirectional long short-term memory network and used ReLU as activation function | [35] |
| Long short-term memory network, their outputs are densely connected to a layer of ReLUs | [40] |
| Long short-term memory network with 3 layers with 10 hidden nodes and use hyperbolic tangent and hard sigmoid as their inner activation functions | [41] |
| Hierarchical clustering is used based on the node connectivity in the interaction graph to reduce the number of nodes by half after each convolution. A max pooling is then performed on each resulting cluster. At the D20ast layer, the remaining nodes are concatenated together and fed into a linear layer to make the final prediction | [42] |
| 1. Convolution on graph, 2. Pooling, 3. Convolution on graph, 4. Pooling, 5. Relation network is used to learn the complex association between graph node groups, 6. Fully connected NN, 7. softmax | [43] |
| 3 hidden layers (one for auto-encoder with L1 regularization), back-propagation | [44] |
