# Supplementary material for: A systematic review of the application of machine learning in the detection and classification of transposable elements
Source: PeerJ. 2019 Dec 18;7:e8311. doi: 10.7717/peerj.8311 (PMC6967008; doi:10.7717/peerj.8311)
Supplement: Supplemental Information 3 [file peerj-07-8311-s003.docx]

**Statement of Rationale and Contribution of the work.**

**Systematic review of the application of Machine Learning in the detection and classification of transposable elements**

**1. The rationale for conducting the meta-analysis**

It is well known that transposable elements (TEs) have many key functions within genomes. They make up most of the genomic information of most eukaryotes, such as in plants and humans. In recent years, numerous algorithms and bioinformatics techniques have been developed with the aim of detecting and classifying this type of sequence. However, none of these methodologies has obtained reliable results due to the complexity of the characteristics of the TEs. On the other hand, Machine Learning has been used in many genomic problems such as genome assembly, detection of genomic variations, association studies of the entire genome and in silico annotation of coded genomic loci. In addition, Deep Learning architectures have shown promising results in huge and complex data such as DNA and RNA, due to their ability to extract characteristics in a nonlinear and automatic way. However, the use of the Machine Learning and Deep Learning approaches in the detection and classification of TE is still limited. This paper systematically reviews the literature to identify the techniques of Machine Learning and Deep Learning successfully applied to the detection and classification of TEs and also in similar data and how their use can improve the reliability of the results obtained by conventional bioinformatics. To our knowledge, this is the first systematic review of the literature focusing on the use of Machine Learning and deep learning in TEs.

**2. The contribution that the meta-analysis makes to knowledge in light of previously published related reports, including other meta-analyses and systematic reviews.**

Following the Literature Systematic Review process, it is possible to demonstrate that the use of Machine Learning (ML) and Deep Learning (DL) for TE analysis (detection and classification) remains an open problem, even though ML and DL have improved outcomes in other genomic problems. In addition, we showed how other researchers demonstrated that the use of labeled data (supervised learning) can generate computational models that are better than current bioinformatics algorithms. These models can be improved by using more data, which will be generated in the coming years by massive sequencing projects. Another benefit of DL over conventional bioinformatics is that DL can extract features automatically. In the specific case of TEs, as they are DNA sequences, the extraction of characteristics is often a too complex process due to the large amount of information, its unstructured form and its sequence. In this case, deep neural networks provide new features that cannot be extracted manually. Finally, in order to propose new ways of using ML and DL in TEs, we summarize some ML and DL architectures and algorithms used in data very similar to those of TEs, which would be extrapolated to these elements.
